# Identification of functional modules based on transcriptional regulation structure

**DOI:** 10.1186/1753-6561-2-s4-s4

**Published:** 2008-12-17

**Authors:** Etienne Birmelé, Mohamed Elati, Céline Rouveirol, Christophe Ambroise

**Affiliations:** 1 Laboratoire Statistique et Génome, UMR CNRS 8071, INRA 1152, Tour Evry 2, F-91000 Evry, France; 2 LIPN – UMR 7030 CNRS – Université Paris 13, 99 Av. J.B. Clément, F-93430 Villetaneuse, France

## Abstract

**Background:**

Identifying gene functional modules is an important step towards elucidating gene functions at a global scale. Clustering algorithms mostly rely on co-expression of genes, that is group together genes having similar expression profiles.

**Results:**

We propose to cluster genes by co-regulation rather than by co-expression. We therefore present an inference algorithm for detecting co-regulated groups from gene expression data and introduce a method to cluster genes given that inferred regulatory structure. Finally, we propose to validate the clustering through a score based on the GO enrichment of the obtained groups of genes.

**Conclusion:**

We evaluate the methods on the stress response of S. Cerevisiae data and obtain better scores than clustering obtained directly from gene expression.

## Background

An important step in analyzing gene functions is to cluster genes according to their expression patterns. Such clusters can then be analyzed in several ways, for example by assigning unannotated genes to the majority function of each cluster's genes (see [1] for a review).

However, this approach has several limitations. On the one hand, genes of similar expression patterns may not necessarily have the same or similar functions; on the other hand, genes with related functions may not show close correlation in their expression patterns. For example, a transcription factor can activate some genes and repress others in the same pathway.

The principal assumption of this paper is that unsupervised clustering of genes on the basis of similar regulators (activators/inhibitors) should assemble functional co-regulated groups of genes. To compute a similarity measure between genes as a function of inferred regulators of these genes, we use the output of a data mining algorithm called LICORN [2], that infers cooperative regulation relations from expression data only. The resulting similarity matrix between genes is considered as the adjacency matrix of a weighted graph. Clustering is then performed to find functional modules of genes in the network.

To objectively evaluate clustering, we use Gene Ontology to determine if the obtained clusters can be associated with terms of the Biological Process ontology. The strength of such an association is given by a *p*-value from an Hypergeometric test. We compare different clusterings by calculating a score based on the *p*-values which becomes greater when the significant *p*-values are smaller and more numerous.

In section Methods, we introduce our model of gene regulation and briefly describe a data mining algorithm for inferring large-scale cooperative gene regulation. We then propose a similarity measure for the genes based on the inferred regulator sets and define the score of a clustering. Finally, in section Results and discussion, we evaluate our system on a yeast data set.

## Methods

### Cooperative regulation networks

**Figure 1:**
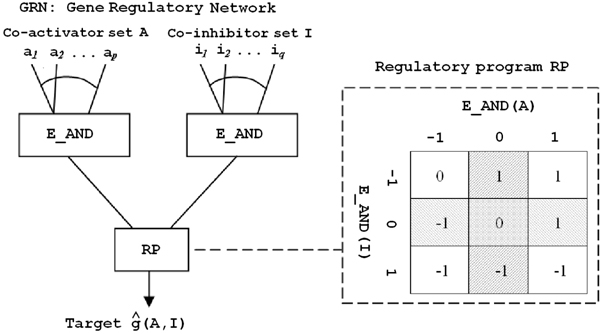
**The regulatory program**. Definition of the regulatory program RP, which can be interpreted as follows: i) If GRN contains co-activators only, $\hat{g}$(*A*, *I*) corresponds to the aggregated status of these co-activators. ii) If GRN contains co-inhibitors only, $\hat{g}$(*A*, *I*) is the inverse of the aggregated status of these co-inhibitors. iii) Otherwise, $\hat{g}$(*A*, *I*) depends on a combination of the statuses of co-activators and co-inhibitors, as described by the matrix on the right. For example, $\hat{g}$(*A*, *I*) = 1 when the co-activators are over-expressed and the co-inhibitors are not.

Let us denote by R the set of genes with a known or putative regulation activity and G as the set of genes without such an activity. The input of the mining method is a discretised expression matrix for genes of R∪G. Each expression value can take the value -1 (under-expressed), 0 (normal), or 1 (over-expressed). A gene regulatory network (GRN) associated with a target gene *g *is a pair (*A*, *I*), where A⊆R is a co-activator set, and I⊆R is a co-inhibitor set. The set of GRNs for all target genes can also be seen as a bipartite graph where the top layer contains regulators, the bottom layer contains target genes, and edges code for a regulatory interaction between regulators and target genes, each edge being labelled with a regulatory mode (i.e., *activator *or *inhibitor*). The regulation relations we are interested in are combinatorial: each target gene has a number of activators and/or inhibitors. Activators on one side and inhibitors on the other side are aggregated in our model through an extended logical AND, i.e., a regulator set *S *(activator or repressor) is over-expressed (resp. under-expressed) if and only if all the regulators in *S *are over-expressed (resp. under-expressed). Finally, we describe in Figure 1 a discrete function called *Regulatory Program *RP, which, given the combined states of activators *A *and inhibitors *I *of *g *in a sample *s *computes $\hat{g}$_*s*_(*A*, *I*), the estimated state of *g *in *s*. The main features of our regulation model are therefore the explicit representation of activation and repression relationships for a given target gene, and the representation of co-operative transcriptional regulation.

### Learning algorithm

We have recently proposed [2] an original, scalable technique called LICORN for deriving co-operative regulations, in which many co-regulators act together to activate or repress a target gene. LICORN uses an original heuristic approach to accelerate the search for an appropriate structure for the regulation network. It first computes frequent co-regulator sets, i.e., regulator sets that frequently occur together as over (1) or under (-1)-expressed in the discretised expression matrix. This is done by using an extension of the Apriori algorithm [3] to handle both 1 and -1 supports (The x-support of a co-regulator *C *in the three-valued expression matrix is the set of samples that include all the regulators of *C *with the state *x*).

From this representation, a limited subset of candidate co-regulator sets is then associated with each gene. The learning algorithm looks for each gene for regulator sets which have a high "overlap" with the target gene. Intuitively, the overlap constraint checks the size of the intersection between supports of the target gene and a given candidate co-regulator set. A candidate activator set for a target gene *g *is frequently over-expressed when *g *is over-expressed or frequently under-expressed when *g *is under-expressed. On the opposite, a candidate repressor set for a target gene *g *is frequently over-expressed when *g *is under-expressed and vice-versa. This search can be efficiently performed because of the property of anti-monotonicity of the overlap constraint with respect to set inclusion. Then, once a limited number of candidate activator and inhibitor sets have been obtained, exhaustive search for the best gene regulatory network can be performed. Finally, a permutation-based procedure is used for selecting statistically significant regulation relations. We have shown in [2] that the co-operative regulation patterns inferred by LICORN cannot be identified by clustering or pairwise methods, and are only partly revealed by constrained Bayesian or decision tree-based techniques, such as those used in previous studies [4,5].

### Identification of functional co-regulation modules

Partial overlap of the regulator sets for a set of target genes can be used as an alternative measurement of the distance between genes.

#### Computation of the co-regulation matrix

We design the co-regulation matrix by using a similarity measure defined as follows: let *λ *∈ [0, 1] and (*g*_1_, *g*_2_) be two genes. The similarity between *g*_1 _and *g*_2 _is defined by

$$\phi (g_{1},g_{2})=\frac{\left|A\right|+\left|I\right|+\lambda \left|AI\right|}{\left|TF\right|},$$

where |*A*| and |*I*| are respectively the number of activators and inhibitors of both *g*_1 _and *g*_2_, |*AI*| is the number of regulators which activate one gene and inhibit the other and |*TF*| is the number of transcription factors regulating at least one of the genes. This similarity considers two genes as being far appart (*ϕ*(*g*_1_, *g*_2_) = 0) if they do not share any regulators. Two genes are considered most similar if their set of activators and inhibitors are exactly the same (*ϕ*(*g*_1_, *g*_2_) = 1). In intermediate situations, *λ *represents the weight given to common regulators which have opposite effects.

#### Clustering

To cluster genes from the similarity matrix, we use the MCL algorithm [6,7]. That algorithm, based on the fluxes in a graph, is well suited to weighted graphs and does not require any prior knowledge about the number of clusters. Moreover, it does not require any initial conditions and is therefore reproducible. The inflation parameter of the algorithm is fixed to 1.8, as suggested in [8].

### Mapping to GO-terms

To assess the functional significance of obtained clusters, and suggest putative functions for genes with unknown functions, we calculate the enrichment of gene ontology (GO) [9].

To determine the over-represented GO terms in each cluster, we apply the R package GOstats [10] with a p-value cut-off of 5% and the *biological process ontology*. For each cluster *C*, we obtain a set *T*_*C *_of GO terms over-represented in *C *with a rate of 5% and a set of associated *p*-values {*p*_*t*_, *t *∈ *T*_*C*_}.

We define the score of the clustering by

$$S(\lambda )=\sum_{C,c_{min}\leq \left|C\right|\leq c_{max}}\sum_{t\in T_{C}}-\log(p_{t})\frac{\left|Ct\right|}{\left|C\right|}$$

where *C*_*t *_is the set of genes of *C *associated with the GO term *t*. Parameters *c*_*min *_and *c*_*max *_allow us to avoid clusters that are too small, which don't have a biological meaning, as well as too big ones, which don't have any functional unity.

## Results and discussion

As a proof of concept, we used gene expression data sets for *S. Cerevisiae*. The Gasch data set [11] measures the response of yeast to 173 stress conditions for 6152 genes. We used a set of 237 known and putative transcription factors.

We applied LICORN and retained only those GRNs (gene regulatory networks) identified as significant with a 5% FDR level (see [2] for details). 2041 GRNs (of 5703 GRNs) were identified as significant. The structural organization of the learned GRNs has been shown to be consistent with recent advances [12] concerning the characterization of topological transcriptional network features in yeast and provide the first evidence of the relevance of inferred GRNs.

**Figure 2:**
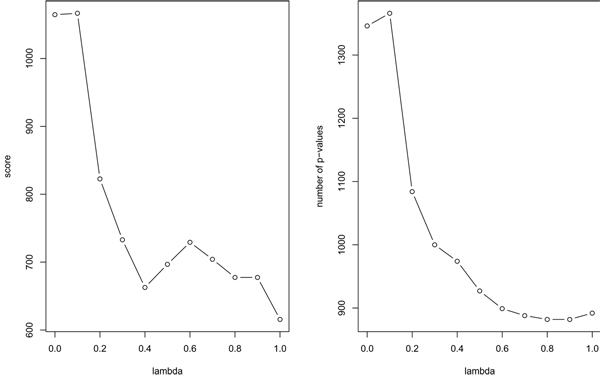
Score and number of *p*-values for *λ *varying from 0 to 1.

In order to choose the parameter *λ *for the similarity matrix, we computed the matrices and the associated clusterings for several values of *λ *and compared their scores with parameters *c*_*min *_= 5 and *c*_*max *_= 200. Figure 2 shows that the best one is obtained for *λ *= 0.1.

For *λ *= 0.1, the clustering gives 30 clusters among which one is too big to be considered (407 genes) and 3 have less than 5 genes. Table 1 gives the best GO term association for the 10 best of them when ranked according to their best *p*-value. The biological evaluation of these clusters is ongoing.

**Table 1:** GO-enrichment of the clusters obtained for *S. Cerevisiae*.

Cluster Id	GO BP Id	*p*-value	Cluster size	Biological process
6	0022613	1.28*e *– 23	80	ribonucleoprotein complex biogenesis and assembly

15	0006119	3.26*e *– 13	18	oxidative phosphorylation

9	0042254	3.15*e *– 11	55	ribosome biogenesis and assembly

4	0006081	4.89*e *– 07	142	aldehyde metabolic process

2	0000746	5.89*e *– 07	155	conjugation

7	0007001	9.48*e *– 07	68	chromosome organization and biogenesis (sensu Eukaryota)

13	0006974	4.10*e *– 05	30	response to DNA damage stimulus

27	0008652	2.02*e *– 04	6	amino acid biosynthetic process

10	0046907	3.50*e *– 04	52	intracellular transport

8	0019754	7.84*e *– 04	66	one-carbon compound catabolic process

Table of the ten best clusters among the 59 ones obtained for *λ *= 0.3 ranked by their best association with a GO term. For each of them, the best associated GO term and the corresponding *p*-value are given, as well as the size of the cluster and the biological process associated to the GO term.

The cluster number 15 that appears on the second line of Table 1 is in fact associated to 32 GO terms with a *p*-value lower than 1*e *– 07, most of those terms being related to phosphorylation or triphosphate metabolic process. Moreover, five genes of that cluster belong to the 167 genes having no associated *GO *term, namely the genes YLR296W, YDR215C, YBL044W, YIR040C and YPR027C. All of them appear in the Entrez gene database but without known functions.

**Figure 3:**
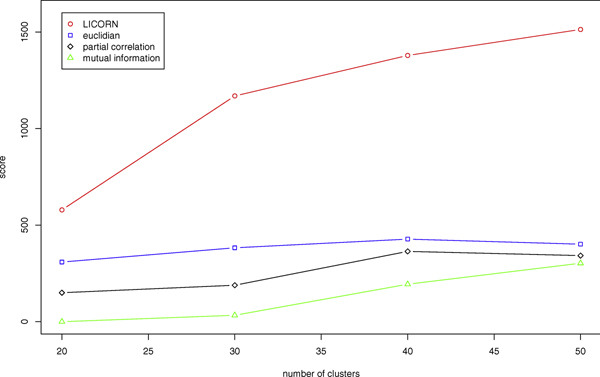
**Comparison of the clustering based on LICORN with existing methods**. Figure of the scores obtained for hierarchical clustering into 20, 30, 40 and 50 clusters. The red circles are the scores obtained for the similarity matrix given by LICORN and *λ *= 0.1. The similarity measures which are compared to are euclidian distance, partial correlation and mutual information.

We have finally validated our method by comparing clustering performances based on other similarity matrices. We therefore have computed from the original expression data matrices of euclidian distance, partial correlation [13] and mutual information [14]. To compare clustering results with the same number of clusters, we used the hierarchical clustering method AGNES [15] to cluster the genes in 20, 30, 40, and 50 groups. Figure 3 shows the scores for those three methods as well as for ours with *λ *= 0.1. It clearly shows that inferring the regulatory network from LICORN preprocessing improves the score of the clustering and provide more biologically relevant clusters.

## Conclusion

The problem of discovering functional modules from expression data is both biologically important and computationally challenging. From a biological perspective, identifying members of functional modules is the first step toward understanding the regulatory network of the cell. We provide here an alternative way for constructing gene modules: genes are clustered in the same module if they share many regulators, as they have been inferred by LICORN from gene expression data. We expect this way of clustering will discover modules that are out the scope of classical co-expression clustering techniques. From a computational perspective, one of the key challenges is dealing with over-fitting as the number of data samples is so small.

## Competing interests

The authors declare that they have no competing interests.

## Authors' contributions

ME and CR designed the inference and participated in experimentations and drafting the manuscript. EB and CA proposed the clustering and evaluation methods.

## References

[B1] Armstrong N, Wiel M van de (2004). Microarray data analysis: from hypotheses to conclusions using gene expression data. Cellular Oncology.

[B2] Elati M, Neuvial P, Bolotin-Fukuhara M, Barillot E, Radvanyi F, Rouveirol C (2007). LICORN: learning co-operative regulation networks from gene expression data. Bioinformatics.

[B3] Agrawal R, Imielinski T, Swami A (1993). Mining Association Rules between sets of items in large databases. Proceedings of the International Conference on Management of Data.

[B4] Pe'er D, Regev A, Tanay A (2002). Minreg: inferring an active regulator set. Bioinformatics.

[B5] Segal E, Shapira M, Regev A, Pe'er D, Botstein D, Koller D, Friedman N (2003). Module networks: identifying regulatory modules and their condition-specific regulators from gene expression data. Nature Genetics.

[B6] van Dongen S (2000). Graph Clustering by Flow Simulation. PhD thesis.

[B7] Enright A, Ouzounis C, van Dongen S (2002). An efficient algorithm for large-scale detection of protein families. Nucleic Acids Research.

[B8] Brohée S, van Helden J (2006). Evaluation of clustering algorithms for protein-protein interaction networks. BMC Bioinformatics.

[B9] Cherry J, Adler C, Ball C, Chervitz S, Dwight S, Hester E, Jia Y, Juvik G, Roe T, Schroeder M, Weng S, Botstein D (1998). SGD: Saccharomyces Genome Database. Nucleic Acids Res.

[B10] Falcon S, Gentleman R (2007). Using GOstats to test gene lists for GO term association. Bioinformatics.

[B11] Gasch A, Spellman P, Kao C, Carmel-Harel O, Eisen M, Storz G, Botstein D, Brown P (2000). Genomic expression programs in the response of yeast cells to environmental changes. Mol Biol Cell.

[B12] Guelzim N, Bottani S, Bourgine P, Képès F (2002). Topological and causal structure of the yeast transcriptional regulatory network. Nature Genetics.

[B13] Opgen-Rhein R, Strimmer K (2006). Inferring gene dependancy networks from genomic longitudinal data: a functional data approach. REVSTAT.

[B14] Meyer P, Kontos K, Lafitte F, Bontempi G (2007). Information-theoretic inference of large transcriptional regulatory networks. EURASIP Journal of Bioinformatics and Systems Biology.

[B15] Kaufman L, Rousseeuw P (1990). Finding Groups in Data: an Introduction to Cluster Analysis.

